# Reversible Speed Regulation of Self‐Propelled Janus Micromotors via Thermoresponsive Bottle‐Brush Polymers

**DOI:** 10.1002/chem.202004792

**Published:** 2021-01-12

**Authors:** Christine Fiedler, Christoph Ulbricht, Tia Truglas, Dominik Wielend, Mateusz Bednorz, Heiko Groiss, Oliver Brüggemann, Ian Teasdale, Yolanda Salinas

**Affiliations:** ^1^ Institute of Polymer Chemistry Johannes Kepler University Linz Altenbergerstraße 69 4040 Linz Austria; ^2^ Linz Institute of Technology Johannes Kepler University Linz Altenbergerstraße 69 4040 Linz Austria; ^3^ Institute of Physical Chemistry-Linz Institute for Organic Solar Cells Johannes Kepler University Linz Altenbergerstraße 69 4040 Linz Austria; ^4^ Christian Doppler Laboratory for Nanoscale Phase Transformations Center of Surface and Nanoanalytics Johannes Kepler University Linz Altenbergerstraße 69 4040 Linz Austria

**Keywords:** bottle-brush polymers, mesoporous silica, micromotors, microparticles, polyphosphazenes

## Abstract

This work reports a reversible braking system for micromotors that can be controlled by small temperature changes (≈5 °C). To achieve this, gated‐mesoporous organosilica microparticles are internally loaded with metal catalysts (to form the motor) and the exterior (partially) grafted with thermosensitive bottle‐brush polyphosphazenes to form Janus particles. When placed in an aqueous solution of H_2_O_2_ (the fuel), rapid forward propulsion of the motors ensues due to decomposition of the fuel. Conformational changes of the polymers at defined temperatures regulate the bubble formation rate and thus act as brakes with considerable deceleration/acceleration observed. As the components can be easily varied, this represents a versatile, modular platform for the exogenous velocity control of micromotors.

## Graphical abstract

**A robust and easily tunable** micromotor platform is reported, based on Janus gated‐mesoporous organosilica microparticles with reversible, external responsive braking systems using bottle‐brush polyphosphazenes to control acceleration/deceleration by small changes in temperature.

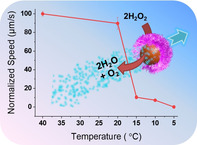

Micro/nanoscale motors ejecting bubbles have attracted considerable attention in recent years for their potential to elicit motion and potentially perform complex operations at the nano/micro scale.^[1]^ Improvements in self‐propelled micro‐ and nanomotors have led to systems capable of motion in different fluids, by utilizing either a physical energy source, such as light or ultrasound,^[2]^ or chemical fuels, such as hydrogen peroxide, water or acids.^[3]^ However, precise movement manipulations, like the control over the velocity and the direction, while particularly desirable, are still limited.^[4]^ Simple velocity control has been reported, for example, by chemical manipulation of systems that poison the catalyst, reducing its effectiveness,^[5]^ or by ion exchange blocking access of the fuel.^[6]^ Alternative systems use local pH^[7]^ or photochemical irradiation to control the speed of propulsion.^[8]^ In a recent example, supramolecular stomatocyte nanomotors decorated with poly(*N*‐isopropyl acrylamide) (PNIPAM) could be directed through temperature variations.^[9]^

As well as the well‐reported polymeric nanoparticles or metal–organic frameworks,^[5]^ micro‐ or nanomotors can also be prepared on the basis of mesoporous hybrid organo‐silica materials.^[10]^ The most common mesoporous type materials with hexagonal porosity arrangement, MCM‐41 (Mobil Composition of Matter) and SBA‐15 (Santa Barbara amorphous material), have been investigated for more than two decades,^[11]^ and allow for a synergetic combination of organic species, silicate and mesopores, surpassing the capabilities of conventional solely SiO_2_‐based materials.^[12]^ Despite their uniform morphology and size, adaptable composition, high‐dense parallel, tubular, unconnected porosity, and exceptional chemical stability and high surface area, the potential use of versatile mesoporous silica materials has not yet been fully explored in the field of micro/nanomotors.^[13]^

Stimuli‐responsive polyphosphazenes acting as molecular gates represent a unique approach among gated mesoporous silica‐based systems, a tactic recently introduced by our group.^[14]^ Their tunable responsiveness,^[15]^ high flexibility, and hydrolytic degradability^[16]^ make these polyphosphazenes outstanding candidates to be utilized in the preparation of stimuli‐responsive micromotors.^[14b]^ Previously, using bottle‐brush polymers as gates, we were able to control the flow in and out of silica particle pores.^[14]^ Herein, we aimed to prepare Janus microparticles based on mesoporous organosilica microparticles (MOMs) capable of operating as self‐propelled, motion controlled micromotors and to empower them with speed regulation by reversibly blocking the mesochannels with stimuli responsive polymers. A common trend in mesoporous silica based micro/nano‐motors fabrication has been to cover one hemisphere of silica particles with catalyst (e.g., Au or Pt) via Janus‐type functionalization.^[17]^ This design exposes continuously the engine to the environment, not allowing switching its off‐on activation at‐will, which limits the catalyst life and possible re‐uses.

Even if that example reported a successful direction control via surface decoration with PNIPAM, there is still room for speed regulation improvements. Therefore, the next step in the motion‐regulation evolution is the incorporation of external stimuli‐responsive controls (off‐on) to easily regulate the movement on demand and stretch the engine function life through the strategical insertion within the pores. We envisaged that opening and closing of the macromolecular gates should essentially control the flow of fuel into and oxygen out of the self‐propelled micromotors and thus act as a braking system. The homogeneous spherical MOMs were prepared according to literature procedures^[18]^ using a 95 to 5 molar ratio of tetraethyl orthosilicate (TEOS) and silane‐functionalized bipyridine (SiBPy) (see synthetic pathway in Figure S6).^[19]^ The synthesized particles ranged between 575 to 700 nm in diameter, exhibiting ca. 3 nm sized pores as determined by (scanning) transmission electron microscopy ((S)TEM), as well as spherical and homogeneous particles size distributions of MOMs, similar to classical mesoporous silica microparticles (MSMs) (see experimental details in the Supporting Information). This was also confirmed by dynamic light scattering measurements (see scanning electron microscope, SEM images in Figure S11, and dynamic light scattering, DLS, together with TEM images in Figure S12). The typical hexagonal and longitudinal porosity found in classical MSM was confirmed for these organosilica microparticles by N_2_ adsorption‐desorption measurements (BET data shown in Table S1) and also clearly observed by TEM (see Figure S12). The MOM particles were extracted by using ethanolic solution of ammonium nitrate, instead of common calcination because of the organic content within the silica framework, giving a surface area of ca. 500 m^2^ g^−1^ and pore volume of 0.66 cm^3^ g^−1^.^[20]^

The content of bipyridine units incorporated was determined by thermogravimetric analysis (1.21 mmol per gram of silica).^[21]^ Then, manganese acetate was added to form bipyridine complexes within the MOM pores (see Figure 1 b).^[22]^ The MOM‐Mn particles were purified and ca. 14 wt % of metal loading was obtained (see experimental details in the Supporting Information). TEM and DLS of the microparticles before and after metal immobilization demonstrated no changes on the textural features or morphology of the microparticles (Figure S12). Furthermore, energy dispersive x‐ray spectroscopy (EDX) mapping by STEM analysis confirmed the homogeneous presence of Mn^2+^ catalyst within the microparticles (see STEM‐EDX in Figures S13 and S14, and quantified EDX sum spectra in Tables S2–3). As expected, the accessible surface area and pore volume decreased drastically after loading the pores with catalysts (11 m^2^ g^−1^ and 0.05 cm^3^ g^−1^) due to the Mn‐bipyridine complex formation in the inner pores.^[22a]^

**Figure 1:**
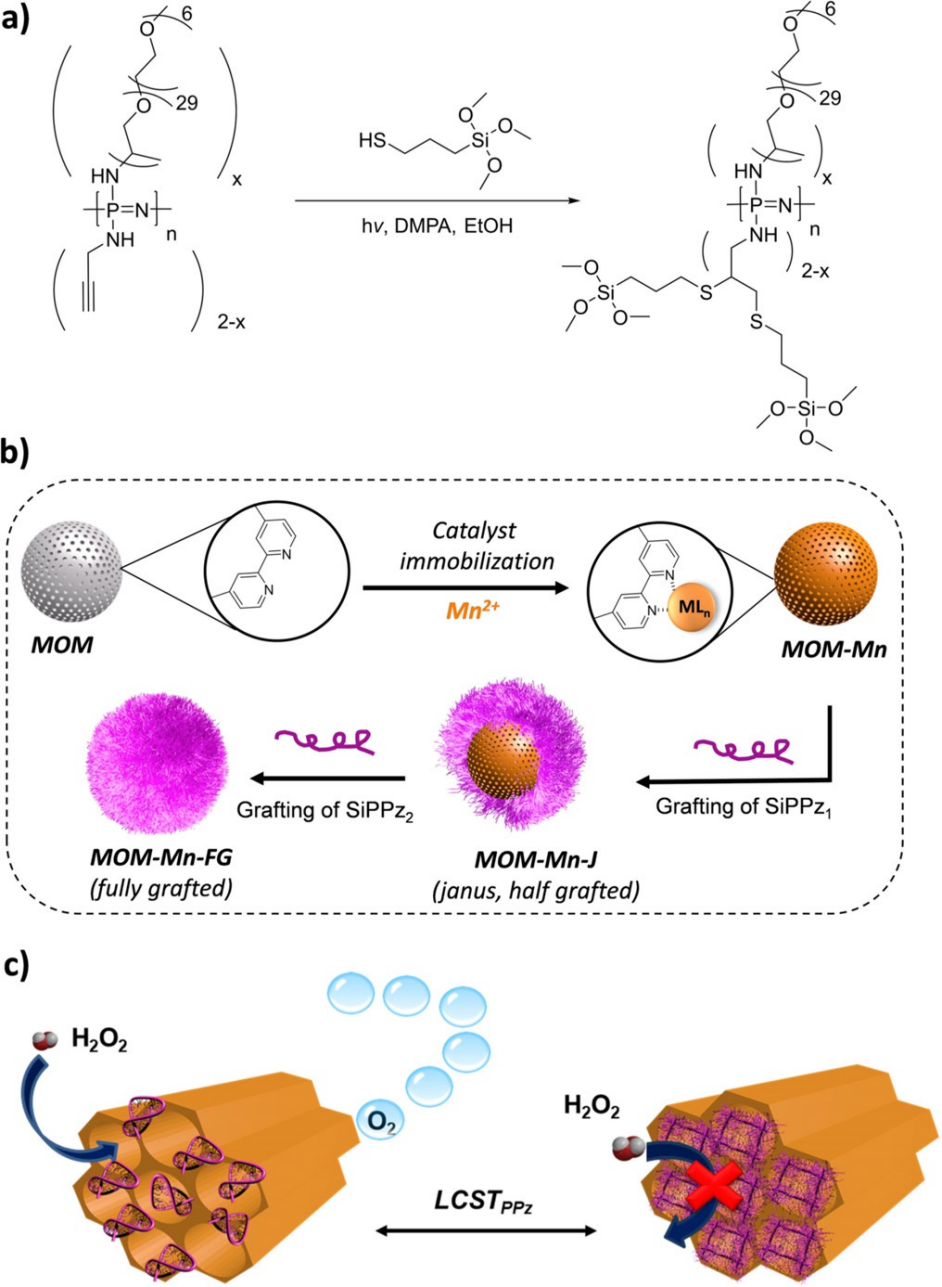
a) Synthesis of trimethoxysilane functionalized polyphosphazenes SiPPz_i_ (n≈50), *x*≈1 or 0.6 (for PPz1 or PPz2, respectively). These substituents are to an extent randomly distributed along the main chain. b) Preparation of Janus and non‐Janus mesoporous organosilica micromotors (MOMs) containing Mn^2+^ immobilized within the pores through bipyridine units (MOM‐Mn), one hemisphere functionalized with SiPPz1 (MOM‐Mn‐J) or fully grafted (MOM‐Mn‐FG). c) Illustration of SiPPz1 on the surface of the catalyst‐loaded pores, arranged in a tubular, honeycomb‐like pattern. The scheme represents the thermo‐responsive conformational changes of the polymeric brushes grafted onto the silica surface.

Separately, two thermoresponsive bottle‐brush polyphosphazenes [NPR_2_]_*n*_ were prepared via phosphine‐mediated living polymerization of Cl_3_P=*N*‐SiMe_3_, carried out according to reported procedures^[23]^ (see synthesis details and characterization in the Supporting Information, Figures S1 and S2). Post‐polymerization functionalization of the resulting [NPCl_2_]_*n*_ with 1 equiv. M‐2005 (a mono amine‐capped polyethylene oxide/polypropylene oxide copolymer, structure shown in Figure 1 a) gives predominantly monosubstituted [NP(R)(Cl)]_*n*_ due to the higher reactivity of the first Cl atom,^[24]^ though some germinal substitution and hence, a randomized distribution cannot be excluded. The remaining chlorine atoms were substituted with a large excess of propargylamine, providing sites for further functionalization. Due to their amphiphilic nature, such macromolecules show a lower critical solution temperature (LCST) behavior in aqueous solutions^[25]^ (see Figures S3–S5, Supporting Information), a temperature above which solubility in aqueous media is drastically lowered due to entropic expulsion of solvated H_2_O molecules and collapse of the solvated coils. The LCST for PPz1 was determined to be around 10 °C (see Figure S5), likewise PPz2. Polyphosphazenes with LCST behavior are well known and applied for example for injectable hydrogels for drug delivery.^[26]^

It is widely reported that the LCST of polyphosphazenes can be easily tuned to the required temperature,^[27]^ and the inherently flexible backbone appears to undergo significant conformational changes upon small changes in temperature. Furthermore, the multifunctionality of the phosphorus units in the polymer main‐chain allows for the addition of multiple functional moieties.^[15]^ Introducing alkyne moieties along the polyphosphazene main‐chain enabled us to incorporate silica binding sites via thiol‐yne addition reaction between the attached alkyne groups and 3‐mercaptopropyl trimethoxysilane. A Pickering emulsion technique was used to coat one side of the silica microparticle by immobilizing them onto wax‐colloids (see scheme in Figure S8).^[28]^ For this, a suspension of MOM‐Mn in ethanol was added to hot wax‐droplets stabilized by cetyltrimethylammonium bromide (CTAB) surfactant to induce the highest surface attachment (see SEM images of the wax colloids with high numbers of MOM‐Mn microparticles immobilized onto the surface, Figure S10). Afterwards, covalent grafting of SiPPz1 to the non‐wax coated hemisphere resulted in Janus microparticles MOM‐Mn‐J (see experimental details in the Supporting Information).^[29]^ The Janus nature and the composition of the particles were confirmed by EDX (Figure 2). The presence of P, S, N, O, Si and Mn elements are shown in Figures S13 and S14 (see values in at. % in Tables S2 and S3). A higher readable signal of P on one of the hemispheres demonstrated the Janus nature of MOM‐Mn‐J (see Figure 2 b), albeit with less than 50 % of polymeric coverage (as observed by STEM‐EDX, Figure 2 a). Significant amounts of nitrogen and sulfur were detected, originating from the bipyridine moieties and the thioether units, respectively. The phosphorus signal was attributed to the surface‐grafted poly(organo)phosphazene chains (see zoomed‐in spectral regions for the elements N, P, S, and Mn in the lowpass filtered STEM‐EDX spectra in Figure S13). Moreover, the remarkable increase in the hydrodynamic diameter (see DLS of MOM‐Mn‐J in Figure S12) was suggestive of polymer grafting on the silica surface.

**Figure 2:**
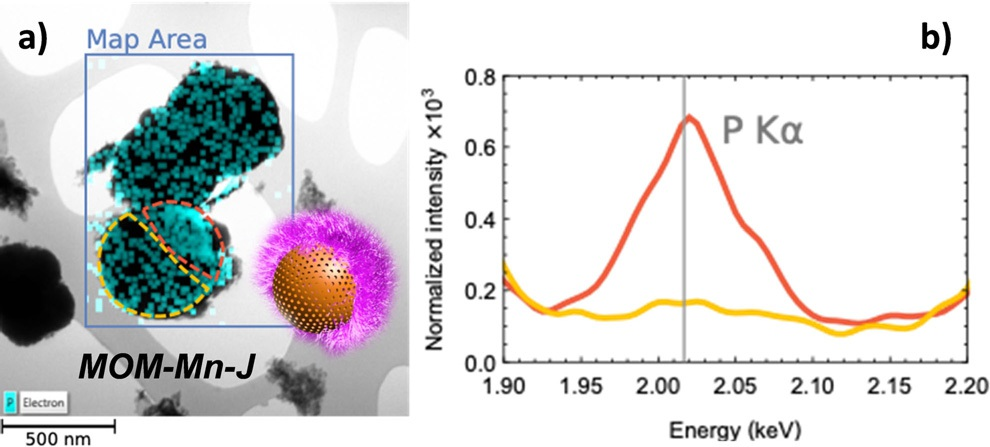
a) TEM image of MOM‐Mn‐J with respective illustration of the micromotor together with STEM‐EDX mapping analysis; b) spectra of MOM‐Mn‐J showing the higher content of P on one side of the microparticle, due to Janus functionalization with polymer SiPPz1.

The Janus particles MOM‐Mn‐J, grafted with SiPPz1, were then suspended in H_2_O. Upon addition of 1 % (v/v) H_2_O_2_ (20 μL) with 1 % SDS, at room temperature a rapid motion of the particles was observed due to decomposition of H_2_O_2_ to O_2_ and H_2_O catalyzed by the Mn^2+^ ions in the MOM cores (see schematic mechanisms in Figure 1 c). The Janus nature of the particles contributed to a forward thrusting trajectory of the particles (see tracking paths in Figure 3 a–b) assisted by the tubular microchannels in the MOMs. In comparison, the fully grafted MOM‐Mn‐FG micromotors showed minimal forward thrusting motion (Figure 3 c–d). This suggests that the grafted polymers do not compromise fuel access in their collapsed conformation, but suffice to force the bubbles preferentially out of the non‐grafted side of the Janus particles. Notably, the speed of the MOM‐Mn‐J particles is extremely much faster than MOM‐Mn‐FG (see values in Table S4), and follows a clear forward thrusting trajectory (see microparticles tracking paths at 5, 20 and 40 °C in Figure S19).

**Figure 3:**
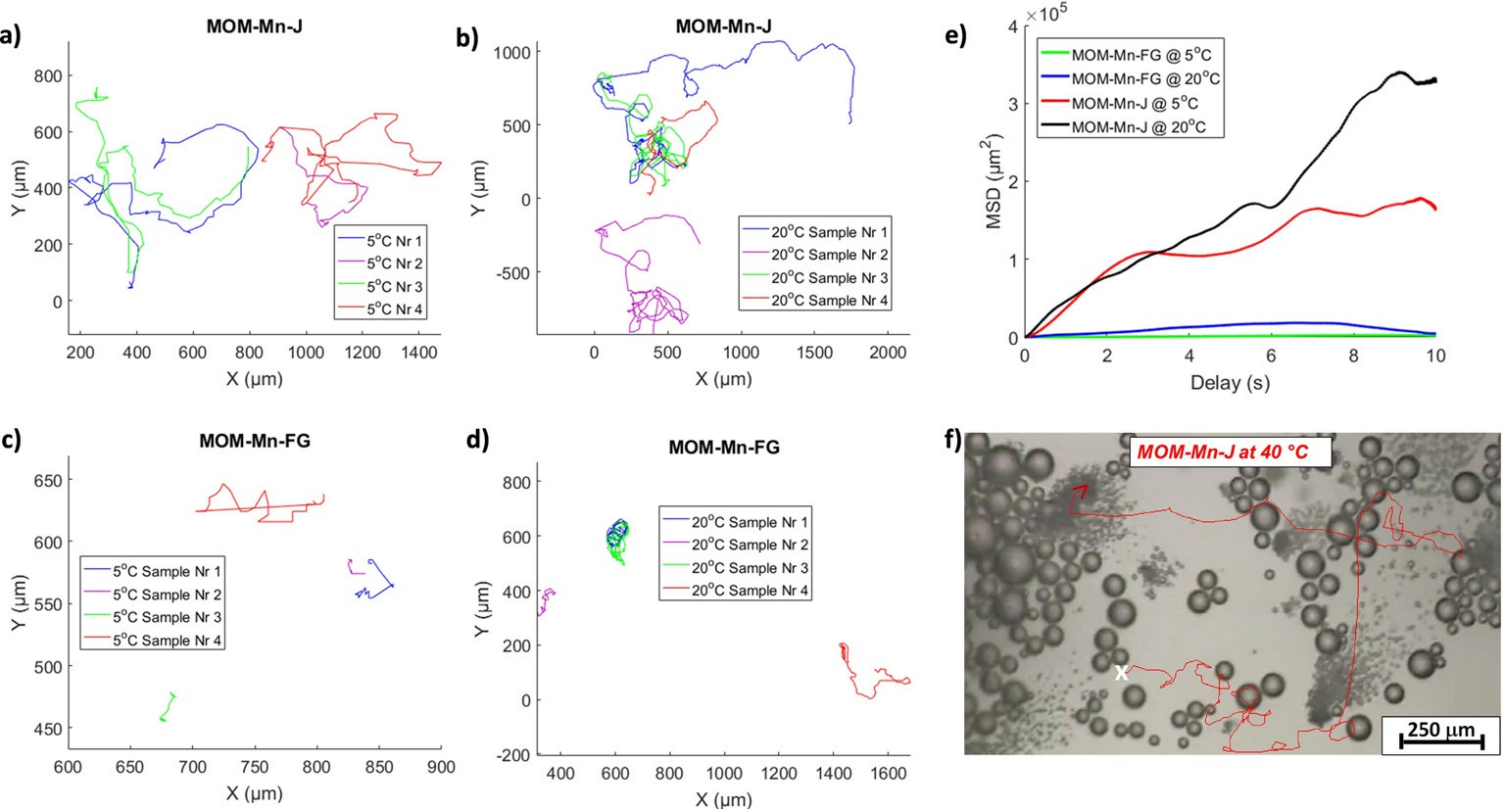
Movement analysis extracted from the optical microscope videos obtained from both half and fully grafted micromotors at different temperatures: Tracking paths of four different microparticles recorded for 10 s from the forward thrusting a–b) Janus micromotors (MOM‐Mn‐J) and c–d) of the fully grafted micromotors (MOM‐Mn‐FG) with temperatures below LCST_PPz_ (5 °C) and above LCST_PPz_ (20 °C). e) Average MSD calculated from the tracking coordinates of 4 particles. f) Optical microscope image of MOM‐Mn‐J, and the corresponding trajectory of one micromotor particle at 40 °C (far above LCST_PPz_), scale bar: 250 μm (the starting point was marked with an *x* and the arrow indicates the particle trajectory). Fuel concentration: 1 % (v/v) H_2_O_2_ with 1 % (v/v) SDS.

Upon reducing the temperature to 15 °C, a pronounced decrease in velocity was observed. The reason for this is thought to be the partial solvation and then, expansion of the PPz1 upon passing below its LCST thus hindering the flow of H_2_O_2_ into the particles.^[14b]^ Note the LCST of the silanized PPz1 on the surface is around 15 °C, higher than the free PPz1, an effect observed in other similar reported works.^[14]^ Calculated mean‐square displacement (MSD) from the tracking coordinates of four particles (from both type of hybrid micromotors), at temperatures below and above LCST also confirmed this temperature controlled braking effect (see Figure 3 e, Figure 4, and Figure S18). In Figure 3 f, a real tracked motion at 40 °C of the Janus micromotors captured with the optical microscope is shown. The process is illustrated in Figure 5 a–c, demonstrating and absence of control for non‐coated MOM‐Mn (a) but a reversible off‐on behavior for MOM‐Mn‐FG (b). The Janus particles (MOM‐Mn‐J) feature speed control with forward thrusting motion (c). This change in speed was observed to be reversible upon increasing the temperature back above LCST (see video SV1‐2 of MOM‐Mn‐J, Supporting Information). The non‐grafted particles MOM‐Mn showed no significant visible difference in velocity when measured below and above LCST (videos SV4 and SV5). Interestingly, albeit a very different system, the observed response is the opposite to that reported by Wilson and co‐workers,^[9]^ and hence complementary to their materials which decelerated upon increasing temperature above LCST of the PNIPAM grafted polymers.

**Figure 4:**
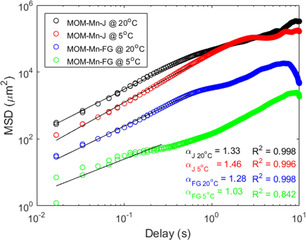
Log‐log representation of MSD fitted with a linear function, for trajectories from MOM‐Mn‐FG and half functionalized Janus‐type MOM‐Mn‐J micromotors at 5 °C and 20 °C.

**Figure 5:**
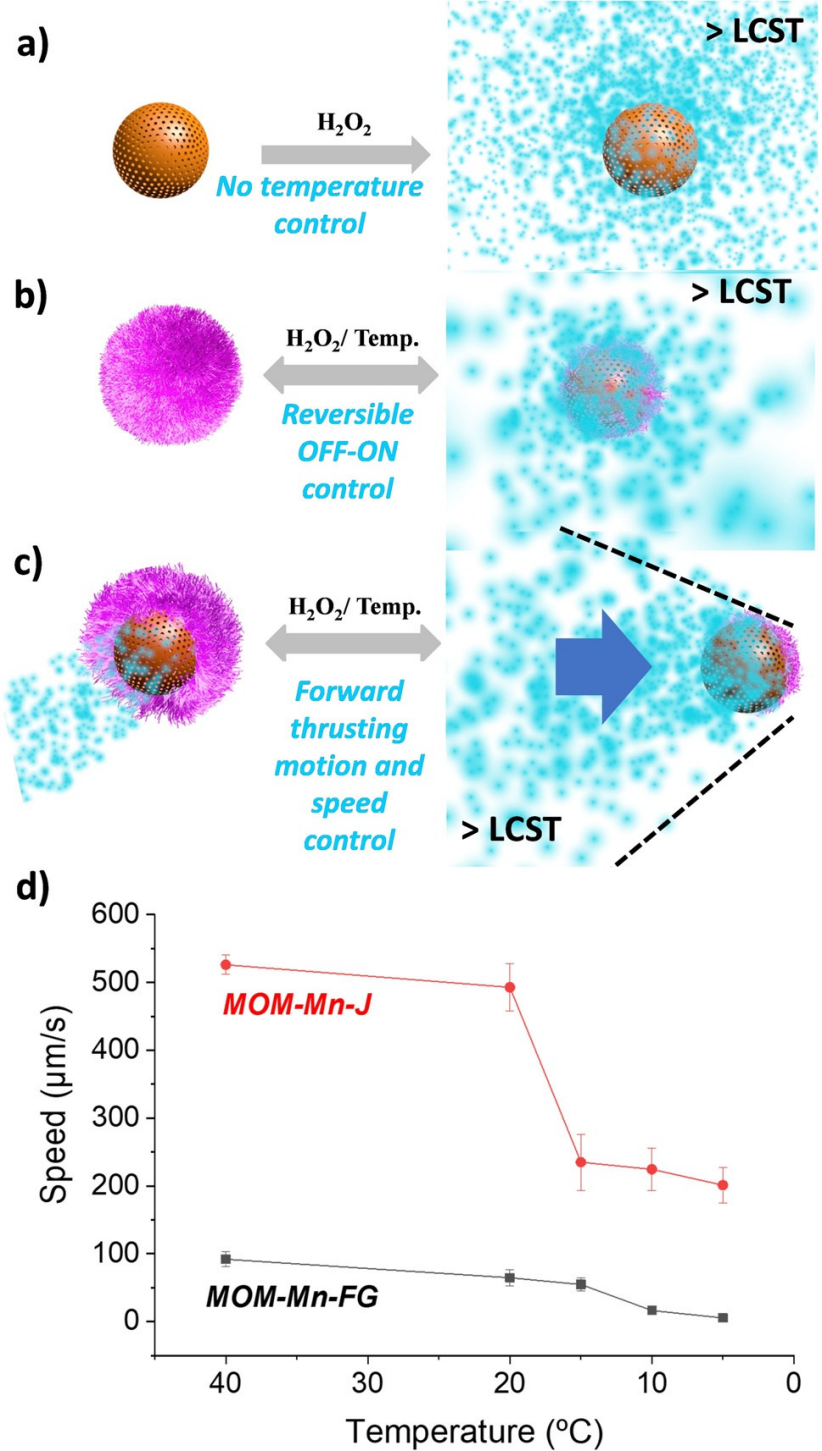
Schematic representation of the possible motion behaviors under temperature changes with H_2_O_2_ as fuel, corresponding to non‐coated MOM‐Mn (a), fully functionalized MOM‐Mn‐FG (b) or half functionalized Janus‐type MOM‐Mn‐J (c) micromotors. (d) Speed graphic for Janus (MOM‐Mn‐J) and fully grafted (MOM‐Mn‐FG) micromotors. Errors calculated from 4 independent speed measurements.

Subsequently, a similar polyphosphazene albeit with slightly more trimethoxysilane groups for binding (SiPPz2) was added to the MOM‐Mn‐J to give fully grafted microparticles MOM‐Mn‐FG (see Figure 1 b and Figure S9). The two‐step approach allowed exactly the same particles to be used as a comparison and it was observed that the MOM‐Mn‐FG particles had a considerably slower velocity than the Janus particles MOM‐Mn‐J (Figure 5 d). Upon reducing the temperature to below the LCST (T≤10 °C) all polymer chains are in an expanded conformation in MOM‐Mn‐FG and the pores are fully closed. Almost no bubble evolution is observed when the pores are closed and thus particle motion was observed to be slowed right down to that of simple Brownian motion (see tracking motion in Figure 3 a–d and speed changes with temperature in Figure 5 d and Figure S17).

The log‐log representation of individual MSD curves vs. time was fitted with a linear function, for trajectories <LCST (at 5 °C) and >LCST (at 20 °C), where all the *R* ^2^ coefficients were >0.8, reflecting good quality of the fit (see Figure 4). The diffusion coefficient (α) was determined from the slope of linear fitted mean square displacement curve at the initial times, from equation log(MSD)=b+α* log(t). According to literature modelling study by Qian et al.,^[30]^ α<1 indicates a motion constrained in space, free diffusive movement for values of *α*=1, while α>1 and close to 2, indicates movement by active transport. Interestingly, at 20 °C (>LCST) both Janus and fully grafted micromotors showed similar values (*α*≈1.3), expected due to the polymer chains collapsing along the whole sphere. Whereas at 5 °C, the fully grafted particles showed values of almost *α*≈1, related to possible constrained or diffusive movement.

The reason for the observed slightly lower transition temperature for MOM‐Mn‐FG is not clear at this present time. Remarkably, we also observed that while the Janus particles MOM‐Mn‐J move in a forward thrusting trajectory, this appears to be lost when the particles are fully grafted (clearly differences shown in MSD analysis, in Figure 3 e). This would further evidence for the aforementioned hypothesis on the O_2_ bubbles evolve predominantly from the non‐grafted side of the Janus particles (see Figures S15 and S16 for the trajectories from the optical motion captured from the microscope, and also the motion behavior of MOM‐Mn‐FG in video SV3).

In conclusion, we have demonstrated the preparation of autonomous, forward thrusting micromotors with a simple braking system which can be switched on by small changes in the environmental temperature. Porous silica particles were loaded with Mn^2+^ as a catalyst and are shown to cause rapid bubble formation upon addition of small quantities of H_2_O_2_ fuel. Grafting of porous silica particles with bottle‐brush PPz in a Janus manner is demonstrated to restrict bubble formation to one side of the particle and thus leads to a forward thrusting motion. Upon reducing the temperature below the LCST of the grafted PPz, a clear braking of the motion was observed in a reversible, switchable manner due to the collapsing/swelling behavior of the polymer brushes. Fully PPz grafted particles showed an almost complete braking of the system, albeit in the absence of forward thrusting motion. The robust nature of the inorganic particles and polymer components and the unique tubular channels of the mesoporous organosilica micromotors, combined with the ease variability of these materials (metal type, particle size, response temperature) represent a versatile, modular platform for the further development of micromotors with exogenous velocity control.

## Conflict of interest

The authors declare no conflict of interest.

## Supporting information

As a service to our authors and readers, this journal provides supporting information supplied by the authors. Such materials are peer reviewed and may be re‐organized for online delivery, but are not copy‐edited or typeset. Technical support issues arising from supporting information (other than missing files) should be addressed to the authors.

Supplementary

Supplementary
