# Supplementary material for: Reversible Speed Regulation of Self‐Propelled Janus Micromotors via Thermoresponsive Bottle‐Brush Polymers
Source: Chemistry. 2021 Jan 12;27(10):3262–7. doi: 10.1002/chem.202004792 (PMC7898474; doi:10.1002/chem.202004792)
Supplement: Supplementary file 2 — Supplementary [file CHEM-27-3262-s002.zip › Supporting Information_ChemEurJ_accepted paper.pdf]

Polyphosphazene preparation

Chemicals used for preparation and characterization of PPz_i, and $SiPPz_i$:

All chemicals and solvents were purchased from commercial suppliers and used as received, if not stated otherwise. Dichlorotriphenylphosphorane (Ph_3PCl_2, 95 %), thionyl chloride ($SOCl_2$, 98 %) and 2,2-dimethoxy-2-phenylacetophenone (DMPA, 99 %) were purchased from Sigma Aldrich. Triethylamine (Et_3N) was purchased from Merck, distilled and dried over molecular sieves (3 Å) before use. 2,2'-Bipyridine-4,4'-dicarboxylic acid(97 %) and propargylamine were purchased from Fluorochem. 3-Mercaptopropyl trimethoxysilane (95 %) was purchased from Alfa Aesar. Jeffamine® M-2005, also known as polyetheramine copolymer (PEO-PPO-NH_2) with a molecular weight of 2000 g mol^{-1}, was purchased from Huntsman Performance Products. Deuterated chloroform ($CDCl_3$), (99.8 %, Sigma Aldrich) and dimethyl sulfoxide (DMSO-d_6 Euriso-Top, 99.8 %) were used for NMR measurements. Solvents were purchased from VWR and Alfa Aesar. Synthesis of monomer ($Cl_3PNSiMe_3$) and poly(dichloro)phosphazene $[NPCl_2]_n$ (n~50) (precursor-PPz) was conducted as reported by our previous paper.[1]

Polyphosphazenes (PPz_i) characterization:

^1H NMR and ^{31}P NMR spectra were recorded at 300 and 121 MHz respectively using a Bruker® Advanced 300 spectrometer. A photochemical reactor from Rayonet with a wavelength of 254 nm was employed for the photochemical thiol-yne addition reaction. Pure water (18 MΩ, MilliQ) was obtained from a Millipore device with a Millipak Express 40 filter (0.22 μm pore size).

Synthesis of PPz_i (n~50) by macromolecular substitution with Jeffamine M-2005 and propargylamine (50:50):

The polymer was created via bulk polymerization where, dichlorotriphenylphosphorane (44 mg, 0.13 mmol, 1 eq.) was dissolved in a small amount of CH_2Cl_2 and placed to stir. Monomer trichloro(trimethylsilyl)phosphoranimine (1.45 g, 6.27 mmol, 48 eq.) was added to CH_2Cl_2 and added dropwise to the mixture and left to stir for > 36 hrs in the glovebox. Once the monomer consumption was complete , Jeffamine® M-2005 (12.65 g, 6.33 mmol) was dissolved in THF (10 mL) and transferred to a flat bottom 250 mL flask and the polymerisation mixture was stirred and added dropwise to the mixture. This was then left to

stir for 12 h. Triethylamine (2.1 mL, 15.10 mmol) and an excess of propargylamine (1 mL, 15.61 mmol) was added to the mixture and the solution was left to stir overnight. The polymer was removed from the glovebox and filtered by gravitational filtration. The flask and the filter residue was washed with additional THF. The filtrate was concentrated, and the remaining material was placed into a vacuum oven at 40 °C for >48 h. Half of the polymer (2.56 g) was dispersed in ethanol and transferred to a dialysis tube (6-8 kD). Dialysis was carried out in water for 3 days with frequent changes of the water (every hour). The dialysis bag with the polymer was transfered into an ethanol bath for 2 days. This was repeated for the remaining half yielding in total 5.07 g of PPz. ^{1}H NMR (300 MHz, $CDCl_3$, δ): 1.09 (s, 90H, -OC**H**$_3$ and -C**H**$_3$ of PPO), 2.18 (s, 1H, C**H**≡C-); 3.31-3.58 (111H, -C**H**$_2$-O -**CH**(Me)- and –C**H**$_2$–C≡C); ppm. ^{31}P NMR (121 MHz, $CDCl_3$, δ): 0.06 (s, –N=PR$_2$–) ppm.

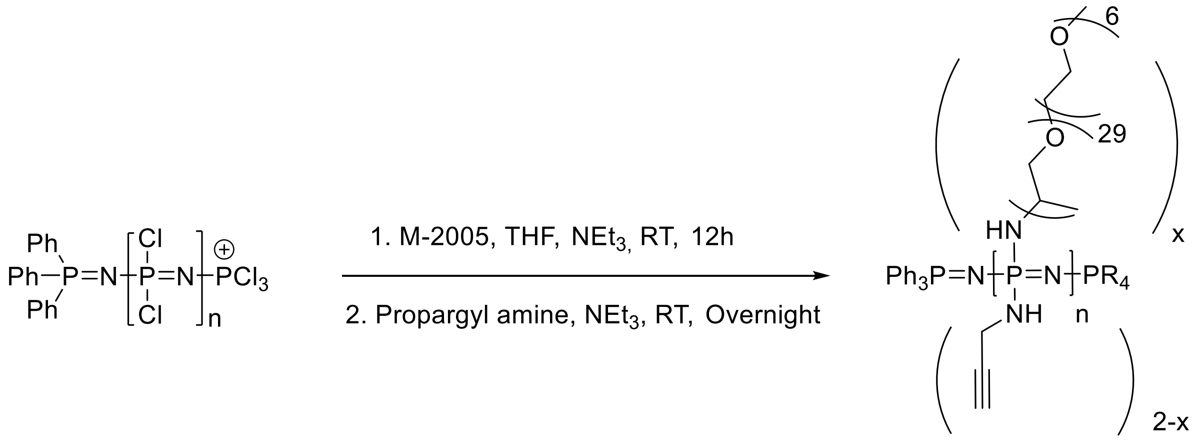

Figure S1. Macrosubstitution with Jeffamine M-2005 to substitute 50% of the chlorine binding sites, followed by a second macrosubstitution with propargylamine in excess to substitute the other ca. 50% of the free chlorine binding sites yielding to PPz1. The scheme of the polymer shows combinations of the two different substituents whereby x ≈1, thus substituents are statistically distributed along the backbone in a ratio of Jeffamine M-2005: propargylamine approximately 1:1.

Synthesis of SiPPz$_i$ by photochemical thiol-yne addition reaction with 3-mercaptopropyl trimethoxysilane:

The polyphosphazene was further functionalized, as reported previously1] PPz$_1$ (1.05 g) was placed in a round- bottom flask and to this photoinitiator DMPA (26 mg, 0.10 mmol) and ethanol (40 mL) were added. The solution was flushed with Argon for 15 mins. After this, 3-mercaptopropyl trimethoxysilane (0.2 mL, 1.0 mmol) was added and the reaction was placed in the UV reactor for 6 h while stirring. NMR spectroscopy of the crude mixture suggested

quantitative functionalization of the silane. The final silane-derived polymer ($SiPPz_1$) was not further purified and directly attached to the silica particles surface. A similar second thermoresponsive polyphosphazene was prepared, called $SiPPz_2$.

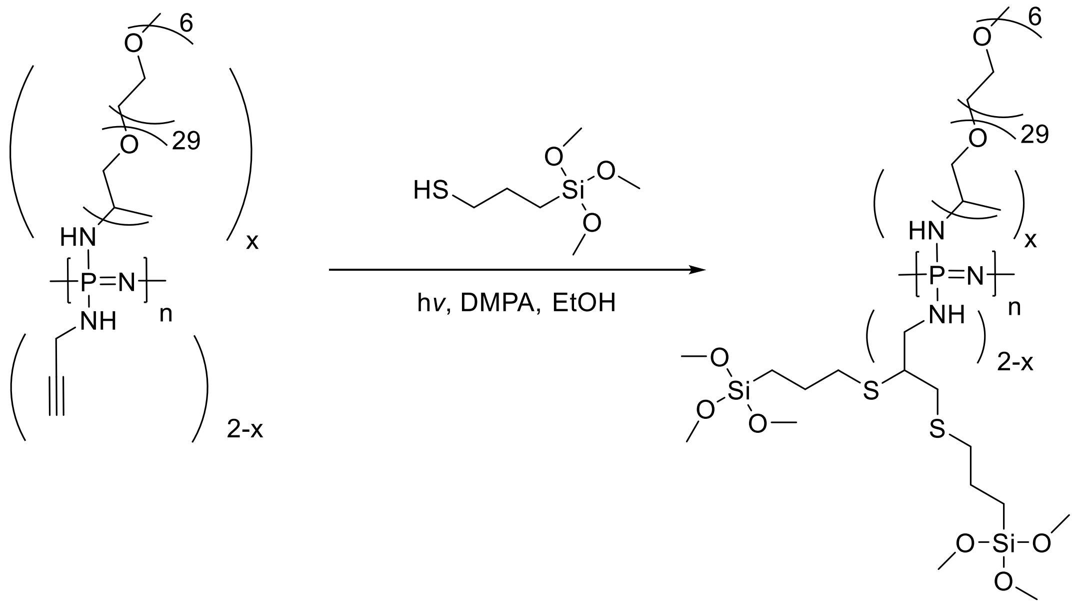

Figure S2. Thiol-yne addition of (3-mercaptopropyl)trimethoxysilane to PPz to yield the silane derived thermoresponsive polymer SiPPz1, (n ~ 50), substituents statistically distributed along the backbone.

Lower Critical Solution Temperature (LCST) determination:

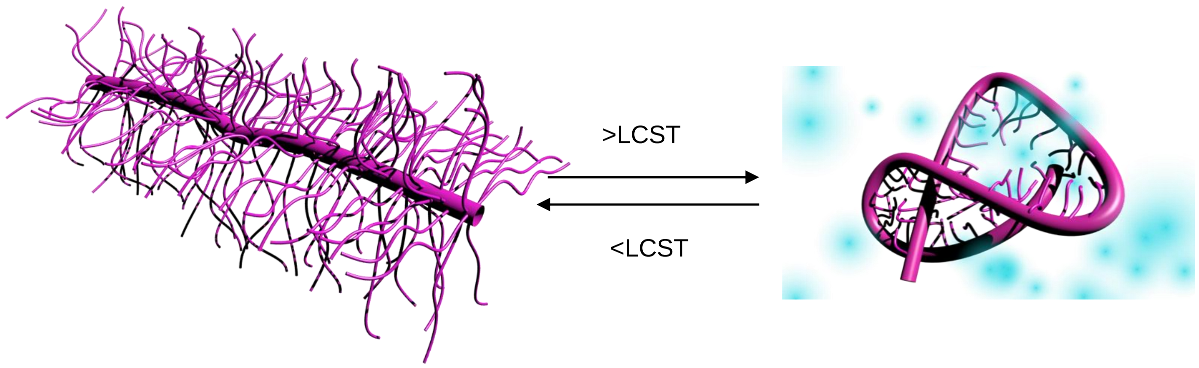

Figure S3. Illustration of the reversible thermoresponsive behaviour of the bottle brush-like polyphosphazene in water, collapsed above the corresponding LCST.

A visual confirmation of the LCST was carried out below and above LCST (at 4 ºC and > 23 ºC (Figure S4) of polymer PPz1 and PPz2 in MilliQ water (1 mg mL^{-1}).

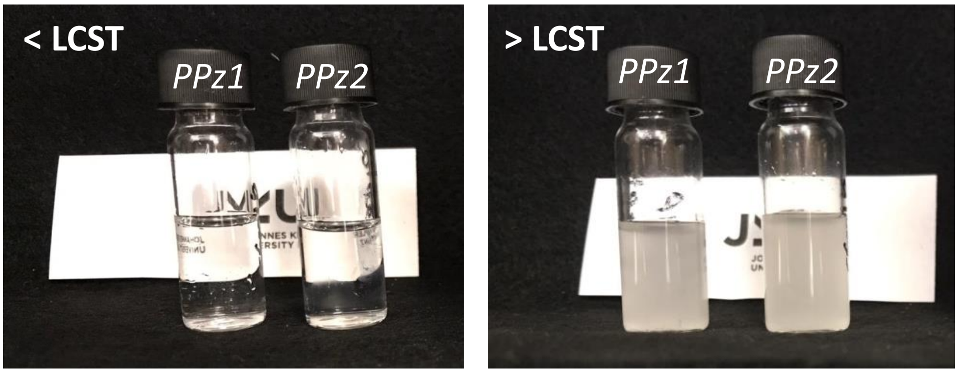

Figure S4. Visual thermoresponsive behaviour of the bottle brush-like polyphosphazene PPz1 and PPz2 in MilliQ water (1 mg mL^{-1}), below and above LCST, at 5 and 25 °C.

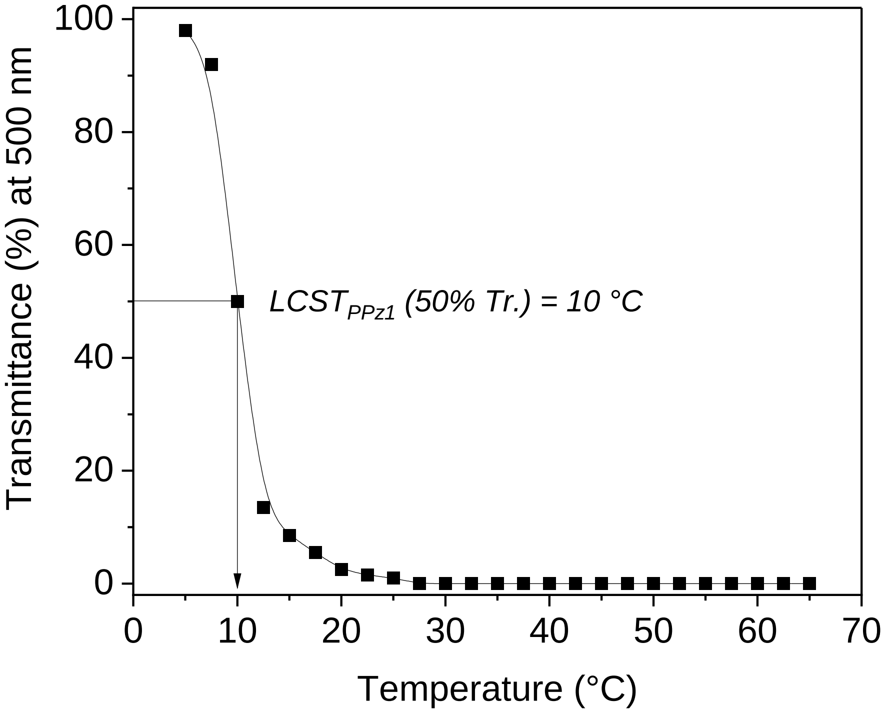

Figure S5. Transmittance (%) measured in a UV-Vis spectrometer at wavelength 500 nm for polymer solution in MilliQ water (1 mg mL^{-1}) varying temperature from 5 ºC to 65 ºC (2 ºC min^{-1}). Lower critical solution temperature (LCST) determined at 50 % of transmittance.

Mesoporous silica microparticles preparation

Chemicals used for mesoporous organosilica microparticles preparation:

Manganese (II) acetate tetrahydrate (> 99.00%) and tetraethyl orthosilicate (TEOS, 98 %) were purchased from Sigma Aldrich. Triethanolamine (TEA, 98%) was purchased from Alfa Aesar. Ammonium nitrate (p.a.) was purchased from Merck. Sodium hydroxide (98 %) was purchased from J. T. Barker. Cetyltrimethylammonium bromide (CTAB, 99 %) and hydrogen peroxide (33 %) were purchased from VWR. Solvents were purchased from VWR and Alfa Aesar. All chemicals and solvents were used as received, if not stated otherwise. Mesoporous silica microparticles were synthetized with deionized water (MilliQ). The bipyridine organosilica precursor (SiBPy) was synthetized as previously reported.[1]

Mesoporous organosilica microparticle characterization methods:

A Perkin Elmer Q 5000 was used for TGA analysis. The samples were placed in platinum pans and measurements were recorded between 50 and 900 °C at a heating rate of 10 °C min^{-1} under a nitrogen flow (25 mL min^{-1}). The BET-BJH data was obtained from a TriStar II 3020 V1.01 with N_2 as an adsorptive in an analysis bath at 77.300 K. Scanning electron microscope images of MSM, MOM and MOM-Mn were obtained using a SEM ZEISS 1540 XB cross-beam scanning microscope equipped with a focused ion beam unit (powder samples without sputtered gold-coating were placed directly ont a conductive carbon tape). In addition, scanning electron microscopy (SEM) images of wax colloids were recorded on a JEOL JSM-6360 LV machine.To this end, microparticles (0.1 mg mL^{-1}) were dispersed in ethanol in an ultrasound bath for 15 minutes. A droplet of the suspension was placed on a flat silicon wafer and left to dry. The samples were sputter coated with a thin gold layer of around 10 nm to reduce charging effects. Images taken by TEM were captured with a Jeol JEM-2200FS at 200 kV. The samples were prepared by using deionized water (MilliQ) as a dispersant (0.1 mg mL^{-1}). After sonication for 15 minutes the dispersed particles were deposited on a 300 mesh copper grid coated with carbon. High-resolution TEM images were captured by zero-loss filtering using an in-column Ω-filter, and further characterization of the elemental composition was carried out by energy-dispersive X-ray spectroscopy (EDX). A zetasizer Nano ZSP (Malvern Instruments) was used to obtain DLS measurements (hydrodynamic diameter). Optical micrographs were recorded with Nikon ECLIPSE LV100ND microscope, equipped with a high-definition color camera head (Nikon DS-Fi2)

and a control unt (Nikon DS-U3). To investigate temperature effects, a setup from Linkam Scientific Instruments, consisting of a compact heating/cooling probe stage (LTS420E-PB4), which was placed on the microscope stage, a system controller (T95-HS LinkPad) with a liquid nitrogen cooling system (LNP95) were used.

*Preparation of classical mesoporous silica microparticles type MCM-41(**MSMs**):*

Non-functionalized MCM-41 microparticles were prepared according to literature.[2] CTABr (508 mg, 1.4 mmol) was dispersed in Milli Q water (480 mL) and NaOH (2mol L^{-1}, 7 mL Milli Q water) in a tall 1 L beaker. The solution was heated to 80 °C for 30 minutes ensuring the pH was basic (ca. 12-13). At the end of the 30 mins, TEOS (10 mL, 44.79 mmol) was added rapidly. The solution was stirred for 2 hrs, and the particles were filtered whilst hot. The resulting microparticles were washed with copious amounts MilliQ water (50 mL) and methanol (25 mL) and dried under vacuum at 40 °C for >12 h (yielding 1.62 g).

*Preparation of mesoporous organosilica microparticles, functionalized with silane derived bipyridine molecule (**MOM**):*

The material synthesis was prepared similarly to that of MSM, however via a co-condensation approach.[3] Silica sourcse TEOS and SiBPy were used in molar ratio 95:5 respectively. Firstly, CTAB (506 mg, 1.39 mmol) was dispersed in Milli Q water (480 mL) and NaOH (2 mol L^{-1}, 7 mL) and stirred at 80 °C for 30 minutes ensuring a basic pH (ca. 12-13). Following this, TEOS (9.5 mL, 42.55 mmol) and silane functionalized bipyridine molecule (2.24 mmol) dispersed previously in ethanol (20 mL) were added. The mixture was stirred for 2 h. After that, the suspension containing the formed silica microparticles was filtered off while still hot and washed with ethanol and water. The microparticles were finally dried under vacuum at 50 °C yielding to a light yellowish powder (2.16 g, MOM with CTAB). In order to remove the CTAB from the pores, the light yellow particles (1.26 g) were extracted by adding to dry ethanol (150 mL) and purified by three cycles of extraction with varying masses of ammonium nitrate as the reflux was carried out at 60 °C for 15-20 mins, then filtered and washed with cold dry ethanol, and repeated. At the end of the last reflux, after filtration and washing, the particles were placed in the oven at 60 °C for 1 hr. A total of 6 cycles were carried out by using 320-380 mg of ammonium nitrate, yielding to a light yellowish powder (**MOM**, CTAB extracted).

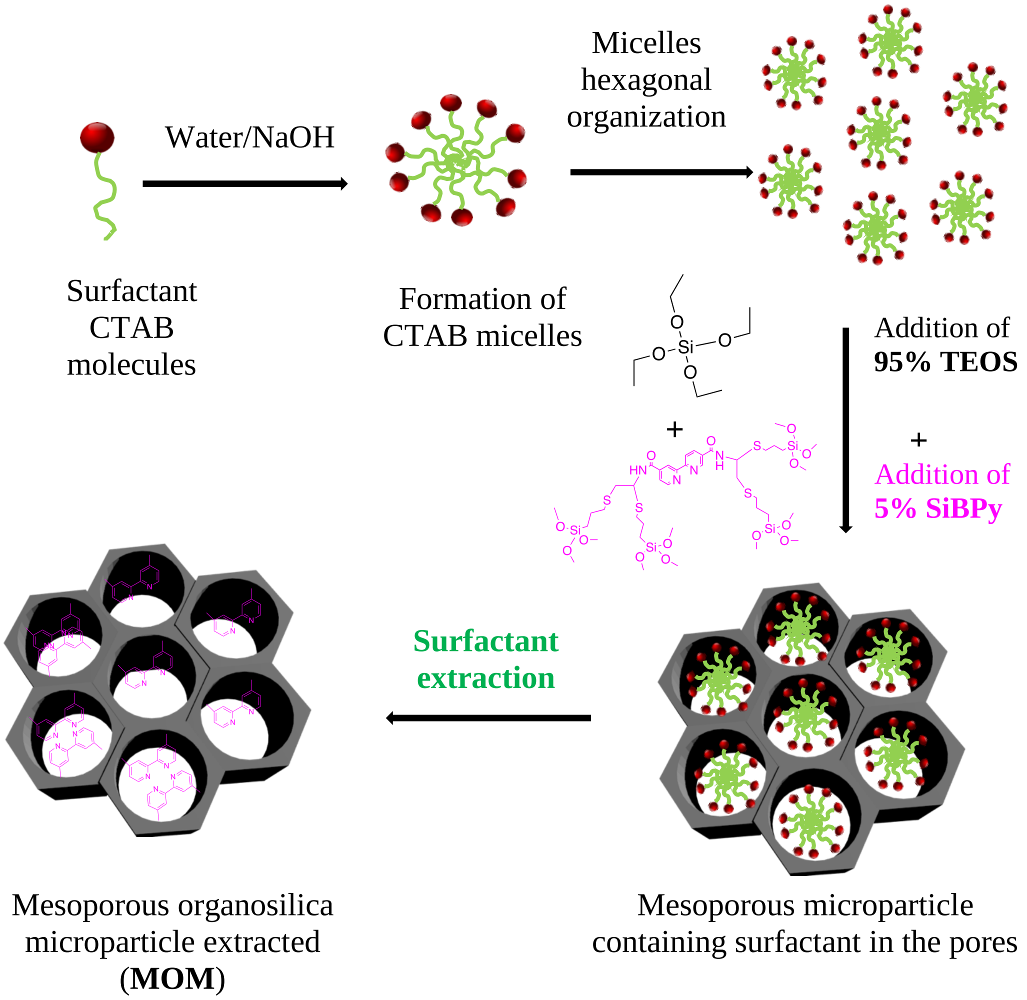

Figure S6. Synthetic pathway for the preparation of mesoporous organosilica microparticles by using 5% silane functionalized bipyridine molecules (SiBPy) and 95 % TEOS, to yield **MOMs** after surfactant ionic extraction with ammonium nitrate in ethanol.

*Manganese immobilization within bipyridine functionalized microparticles (**MOM-Mn**):*

The manganese bipyridine complex formation within the pores from previous prepared BPy-containing microparticles (see Figure S7) was carried out according to literature.[4] This involved manganese (II) acetate tetrahydrate (220 mg) was added to toluene (40 mL) in a round bottom flask, together with the surfactant extracted **MOM** (200 mg) and the mixture was refluxed at 110 °C for more than 12 h under nitrogen atmosphere. The particles were filtered and washed with toluene (40 mL) and acetone (5 %, 10 mL), and dried in the oven under vacuum at 50 °C for more than 12 h (yielding 246 mg of **MOM-Mn**).

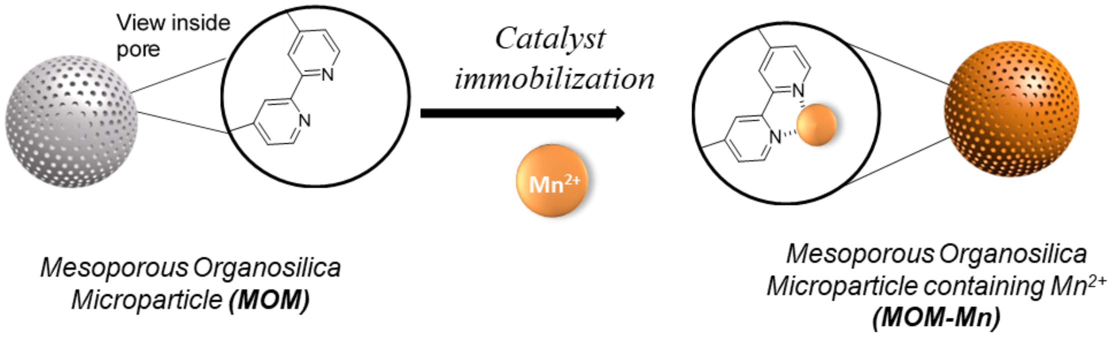

Figure S7. Schematic illustration of the colour change of the mesoporous silica particle after immobilization of Mn^{2+} ions within the silica pores through interaction with bypiridine units.

Preparation of the Pickering emulsion:

In order to functionalize one hemisphere of the silica mesoporous microparticles containing Mn, the MOM-Mn have to be immobilized for this a Pickering emulsion method adapted from literature was used .[5] Firstly, CTAB (2.81 mg) was dispersed in ethanol (15 mL) in a tall vial (30 mL) and whilst stirring the mixture was heated to 75 °C for 10 min. To this, temperature paraffin wax (1.04 g) was added slowly to the solution and was left to stir for 15 mins while keeping the temperature constant (see schematic illustration in Figure S9).

*Preparation of Janus micromotors (**MOM-Mn-J**):*

To fabricate the Janus micromotors, a classical grafting-to approach was taken as already reported.[6] The MOM-Mn particles (220 mg) were dispersed in ethanol (20 mL), sonicated for 5 minutes and stirred under N_2 atmosphere at room temperature. This suspension was then added to the hot wax mixture, and the polymer SiPPz1 (0.53g in 20 mL EtOH) was added. The mixture was left to stir for 24 h. The solution was then allowed to cool slowly to room temperature. The reaction mixture was then filtered and the resulting wax-particles were washed with ethanol and acetone (10 mL) and dried at room temperature for more than 12 h (yielding ca. 900 mg of wax-particles). In order to obtain the one-hemisphere functionalized organosilica microparticles (MOM-Mn-J), the wax was then removed (see Figure S8). For this the microparticles were suspended in n-heptane (40 mL) and placed on the orbital shaker (180 rpm) for >12h. The resulting particles were filtered off and dried at room temperature for 2 h before immersing them in ethanol (20 mL). The solution was placed to stir under N_2 atmosphere at room temperature.

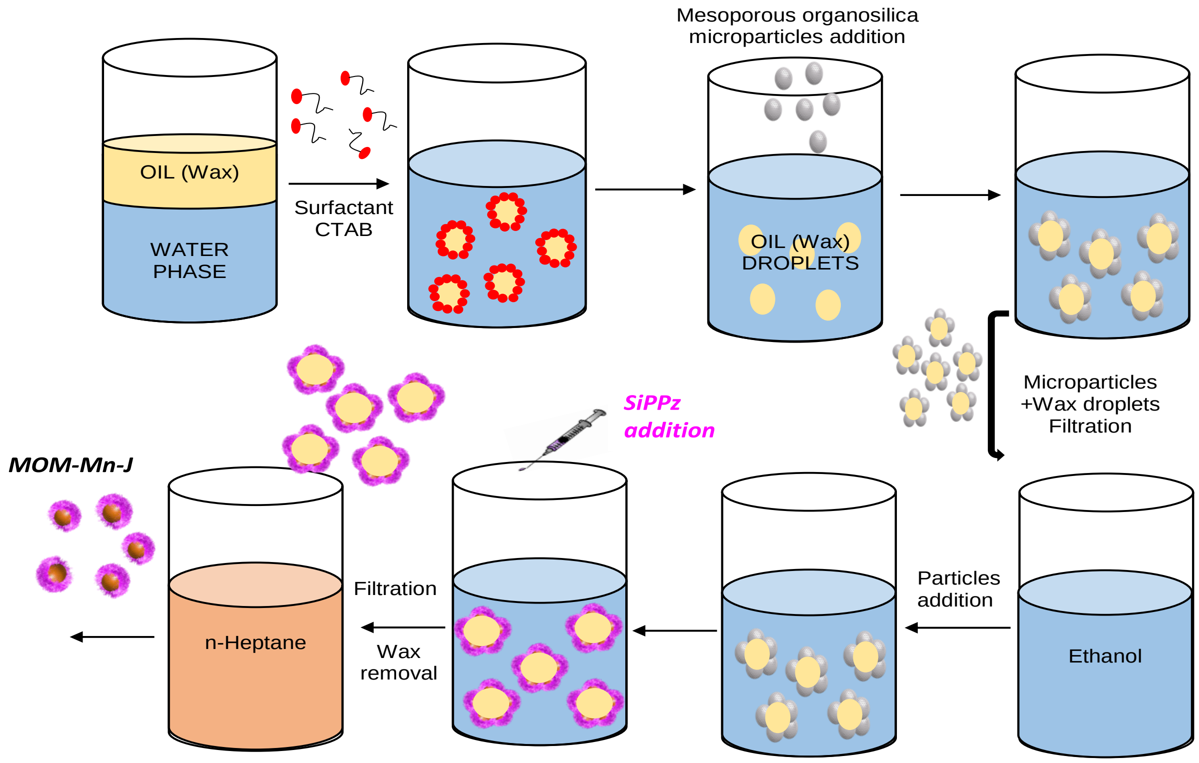

Figure S8. Schematic illustration for preparing one-hemisphere functionalized Janus micromotors (**MOM-Mn-J**) by wax Pickering emulsion method.

*Preparation of fully-grafted micromotors (**MSM-Mn-FG**):*

In order to graft the free 2nd hemisphere of the silica microparticles with polymer as well, previously dried one-hemisphere functionalized Janus microparticles (MSM-Mn-J, 100 mg) and SiPPz2 (0.25 g) were suspended in EtOH (10 mL) and the mixture was left to stir for 24 h (as shown in Figure S9). After this, the mixture was filtered and the resulting particles were left to dry at room temperature >12 h, yielding the final fully grafted motors MOM-Mn-FG.

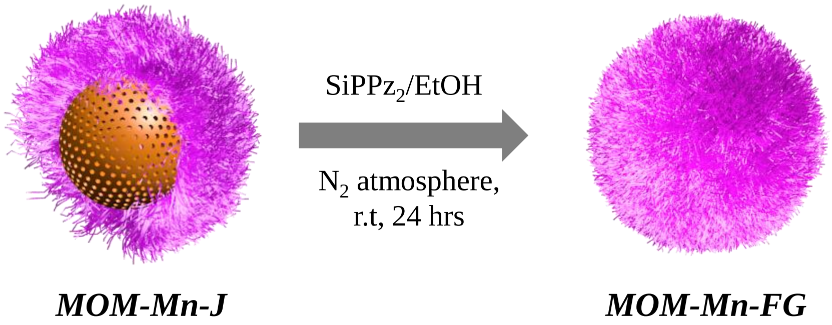

Figure S9. Schematic illustration of fully grafting to the pores surface of Mn-loaded micromotors **MOM-Mn-J** with similar second thermoresponsive polyphosphazene SiPPz2, to yield fully grafted mesoporous organosilica micromotors **MOM-Mn-FG.**

Characterization of the microparticles:

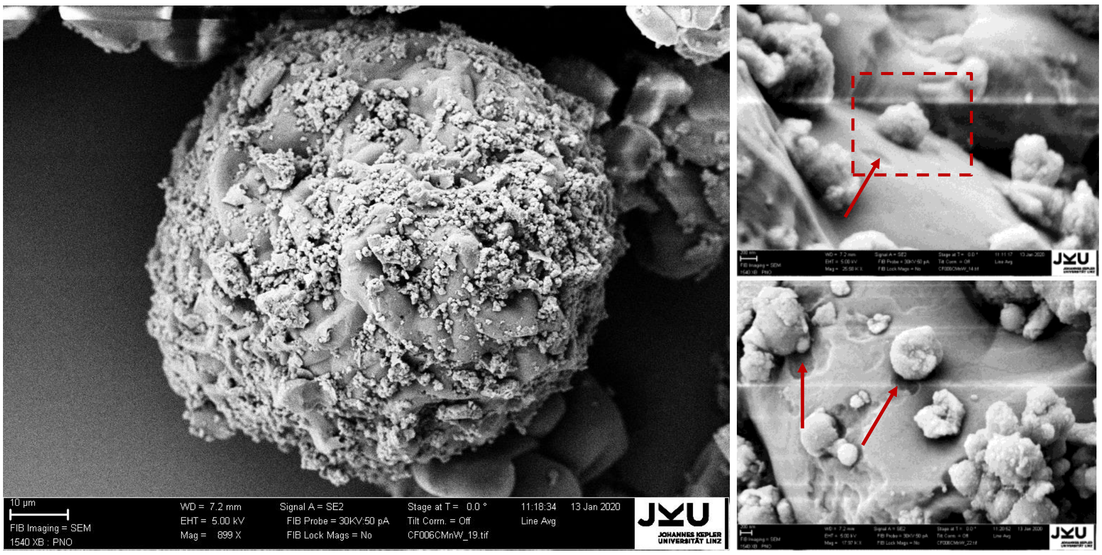

Figure S10. SEM images taken with 5 kV beam energy of the wax colloids formed during the Pickering method, presenting high amount of MOM-Mn immobilized onto the wax surface: a) at 10 micrometers; and b-c) at 2 and 1 micrometer showing a closer view, arrow pointing a cavity left behind after one silica microparticle was detached from the wax colloid.

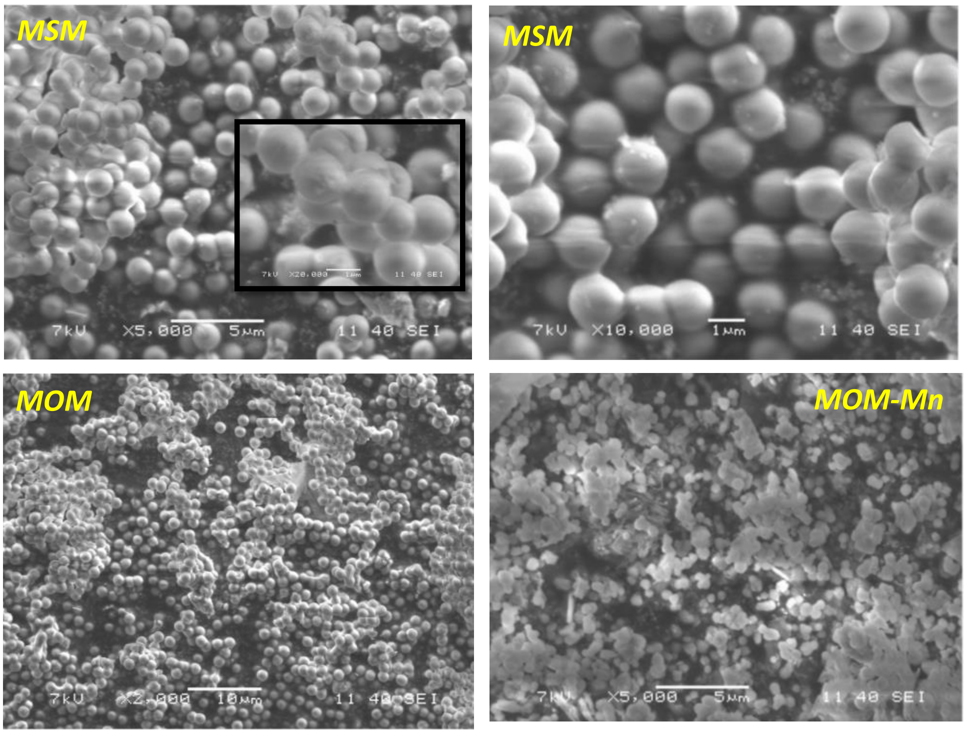

Figure S11. (Top) SEM images taken with 7 kV beam energy of classical mesoporous silica microparticles (MSM) at 5 and 1 micrometer (inset); (Down) bipyridine containing mesoporous organosilica microparticles (MOM) and Mn-loaded organosilica microparticles (MOM-Mn) at a 10 and 5 micrometer scale, showing monodispersed and spherical microparticles.

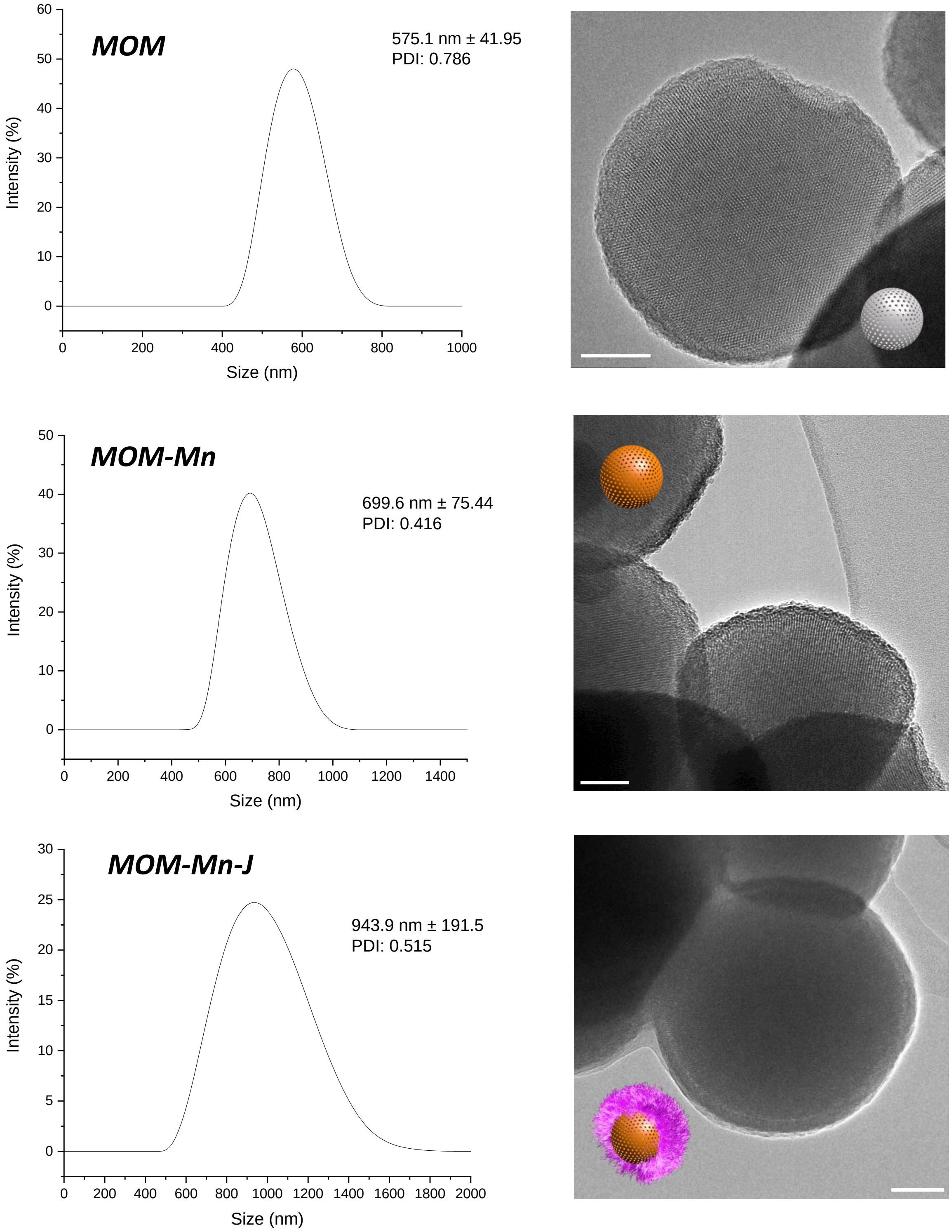

Figure S12. Dynamic Light scattering measurements determining the hydrodynamic diameter and PDI, together with corresponding TEM images, of next micromaterials: MOM, MOM-Mn and MOM-Mn-J, showing the similar spherical shape, and longitudinal, hexagonal ordered porosity (pore size ca. 3 nm). Scale = 100 nm. Pores less visible in MOM-Mn-J due to the presence of the polymer onto the silica surface decreases the TEM image resolution.

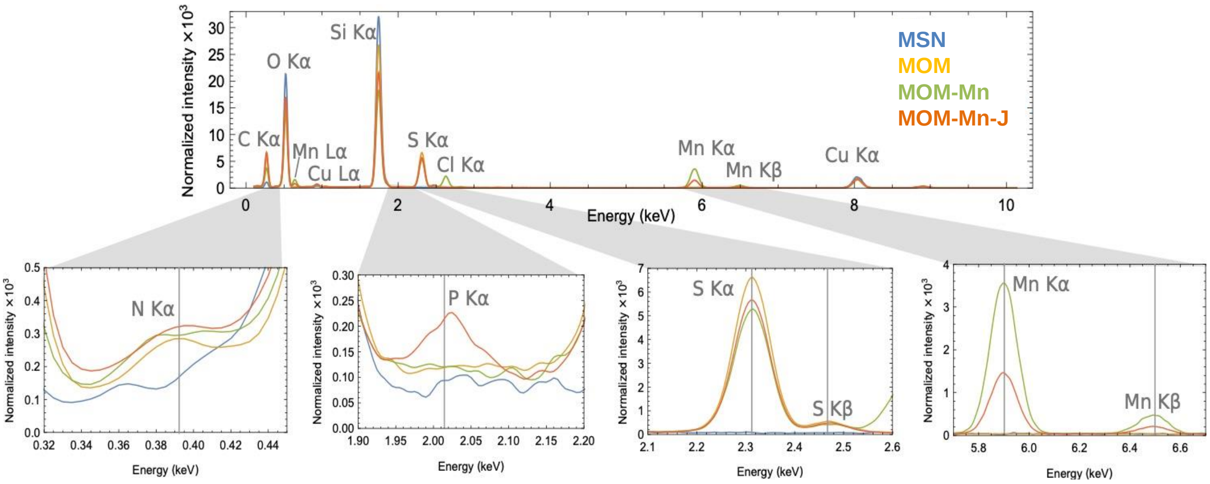

Figure S13. Lowpass filtered STEM EDX spectra of MSM, MOM, MOM-Mn, and MOM-Mn-J. Insets show zoomed-in spectral regions for the elements N, P, S, and Mn.

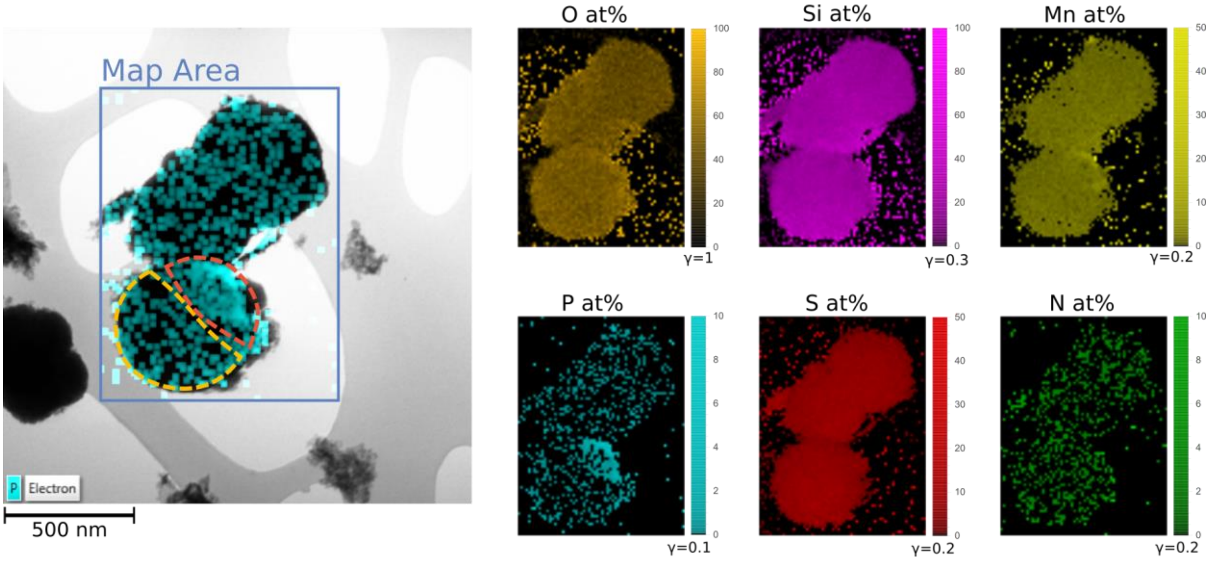

Figure S14. STEM-EDX mapping of the Janus micromotor MOM-Mn-J. The Janus feature could be mapped, if the interface between the two half is parallel to the view of sight, which is the case for one of the three particles

Table S1. Characterization parameters measured from N_2-adsorption–desorption isotherms.

Microparticles	S_{BET} area m^2g^{-1}	Pore vol.[a] cm^3g^{-1}	Pore size[a] nm
MSM *(CTAB extracted)*	609	0.56	2.94
MOM *(with CTAB)*	88	0.34	-
MOM *(CTAB extracted)*	496	0.66	3
MOM-Mn	11	0.05	-

Table S2. Quantified EDX sum spectra (without Cu, C) in at% of microparticles

	N	O	Si	P	S	Mn	Na	K	Cl
MSN	0.0	67.7	32.3	0.0	0.0	0.0	0.0	0.0	0.0
MOM	1.5	57.0	32.7	0.0	8.5	0.0	0.3	0.1	0.0
MOM-Mn	1.4	61.7	22.9	0.0	6.9	4.4	0.0	0.0	2.7
MOM-Mn-J	1.0	64.8	25.6	0.1	7.0	1.7	0.0	0.0	0.0

Table S3. Quantified EDX sum spectra (without Cu, C) in at% for both hemispheres from the Janus microparticle (half 1 and half 2 relate to the marked areas in Figure S14)

	N	O	Si	P	S	Mn	Na	K	Cl
Half1	1.0	65.1	25.6	0.0	6.9	1.4	0.0	0.0	0.0
Half2	1.5	67.5	22.9	0.6	5.6	1.9	0.0	0.0	0.0

EDX sum spectra were obtained by adding up the counts stemming from the microparticles. These spectra were quantified by using a high statistical threshold to differentiate between background noise and elemental peaks. The overall concentrations detected in MSN, MOM, MOM-Mn, MOM-Mn-J are listed in table S2, the corresponding spectra are shown in Figure S13. In order to show the elemental distribution, the spectra are quantified pixelwise. Therefore, the counts collected in each pixel are much lower and the single pixel spectra are highly noisy. The statistical threshold for the quantified mappings had to be lowered. Nevertheless, changes in concentrations which are not due to thickness changes were observed in the P map for MOM-Mn-J (see Figure S14). This behavior could not be detected for all particles, but the bottom particle featured a Janus appearance. To confirm this, the sum spectra of the two hemispheres were extracted (yellow and red marked regions in Figure S14). The P peak is clearly only visible in one of the two halfs (see Figure 2b in the main article). The quantified P concentration in the two hemispheres is given in table S3. Since the concentrations from the functionalization compounds are very low, and EDX is not well-suited for light elements such as P, N and S, the quantified results do not represent the true concentrations. However, the method shows that the presence of the detected elements matches the functionalization steps and the relative difference of P in a Janus microparticle could be reported, which confirmed the one-sided coverage with PPz.

Motion characterization of the micromotors:

Temperature responsiveness studies:

The micromotors were dispersed in aqueous solution of 1 % H_2O_2 (20 μL) and 1% SDS (20 μL) was added onto a microslide. The evolution of oxygen in the form of bubbles was analyzed under an optical microscope with a high definition camera head along with a heating/cooling stage for temperature control. The motion and bubbles formation were recorded and tracked by images and videos at temperature range of 5 °C-40 °C (see Figures S16 and S17).

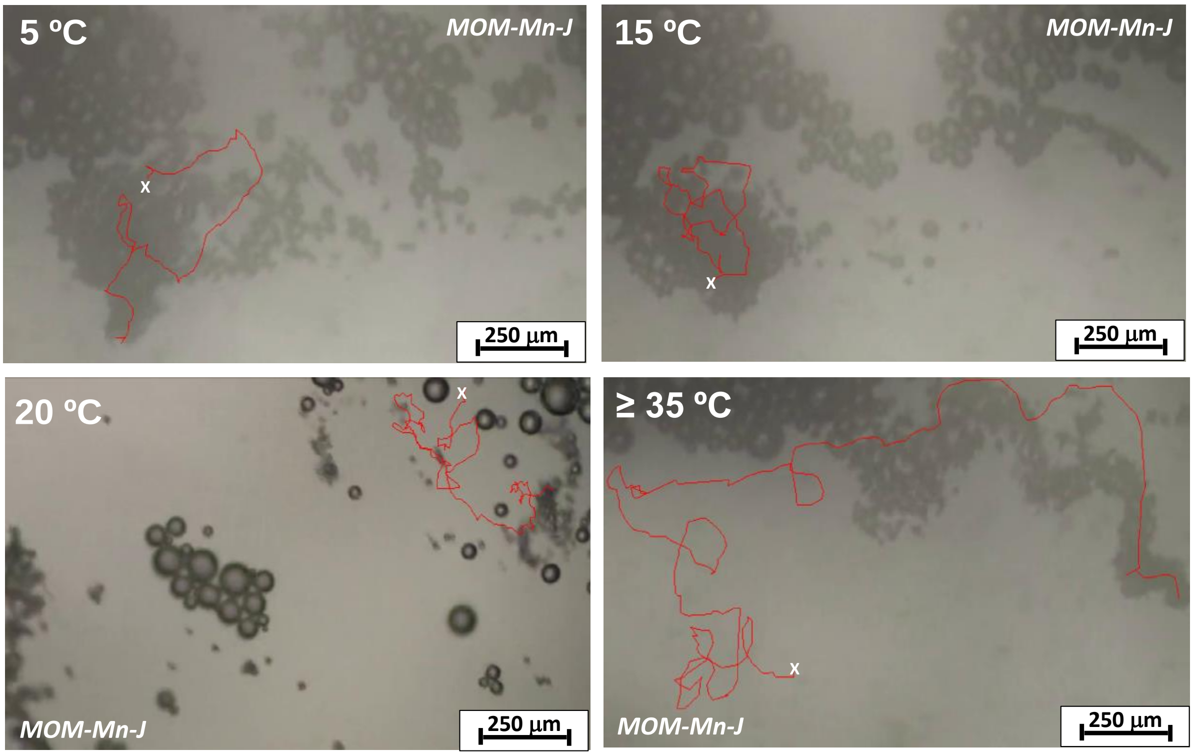

Figure S15. Optical microscope images of MOM-Mn-J, with their tracked motion at different temperatures below and above $LCST_{PPz}$ (x is the starting point of the motion). Scale bars: 250 μm. Fuel concentration: 1% (v/v) H_2O_2 in 1% (v/v) SDS.

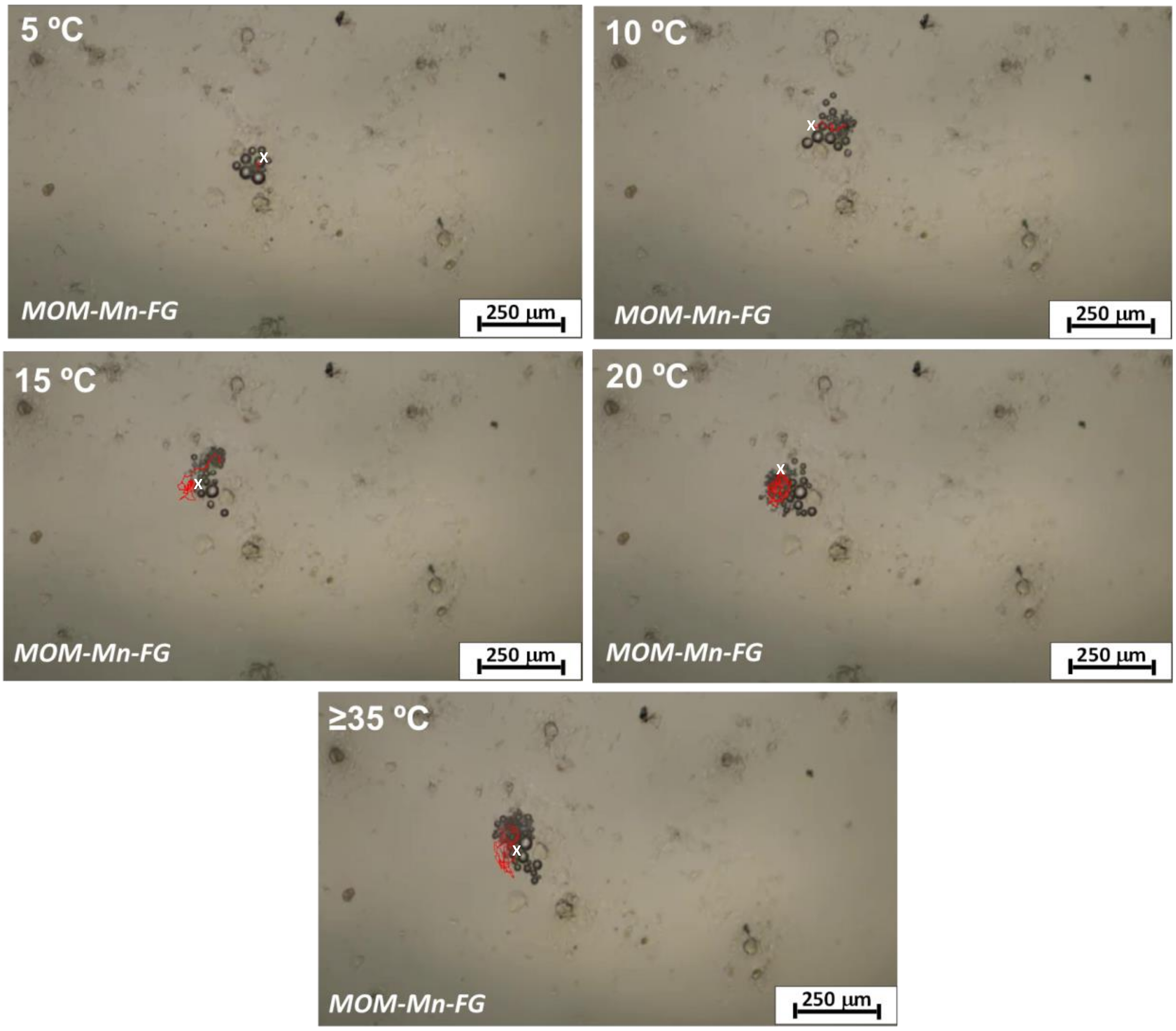

Figure S16. Optical microscope images of MOM-Mn-FG, and their tracked motion at different temperatures below and above $LCST_{PPz}$ (x is the starting point of the motion). Scale bars: 250 µm. Fuel concentration: 1% (v/v) H_2O_2 in 1% (v/v) SDS.

Speed calculations from tracking the micromotors:

A tracking software (the micromotors were tracked with Tracker Video Analysis and Modelling Tool, open source Physics, Douglas Brown version 5.13) was used to track the pathway of the micromotors in motion. The distance travelled by the micromotor was calculated with the scale bar from the image and determined by the magnification error given on the video taken from the optical microscope during 10 s. The speeds calculated were determined for temperatures above LCST (40 °C, 20 °C and 15 °C) and at 10 °C and 5 °C (≤ LCST), data collected in Table S4.

Table S4. Speed values calculated for Janus and fully grafted micromotors.

Errors calculated from 4 independent speed measurements (from four different particles).

Micromotor	Temperature (°C)	Speed (μm s^{-1})
**MOM-Mn-J**	40	528.7 ± 10.9
	20	494.9 ± 24.9
	15	225.1 ± 33.9
	10	217.5 ± 25.5
	5	152.4 ± 27.7
**MOM-Mn-FG**	40	85.4 ± 18.3
	20	63.1 ± 8.8
	15	55.1 ± 6.7
	10	16.5 ± 4.2
	5	5.3 ± 2.4

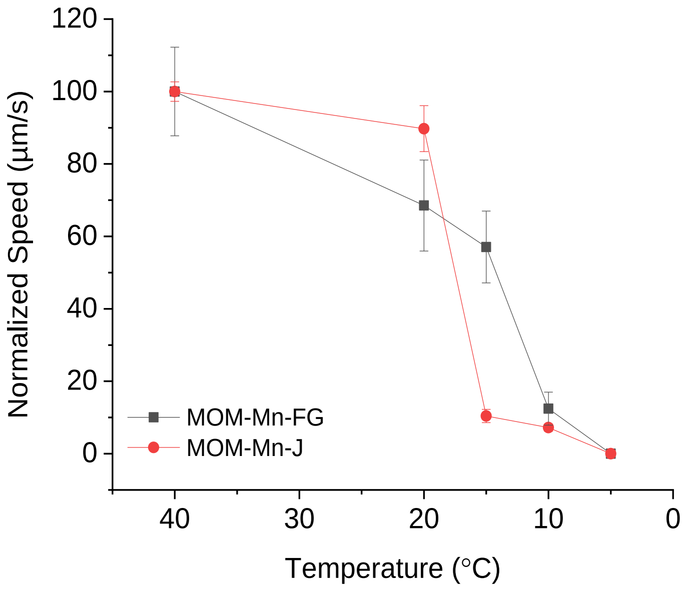

Figure S17. Normalized speed graphic for Janus (MOM-Mn-J, circles symbol) and fully grafted (MOM-Mn-FG, square symbols) micromotors, showing similar switching on thermoresponsiveness, shown above 10 °C. Errors calculated from three independent speed measurements.

Mean square displacement (MSD) analysis:

This method was used as reported from literature[7]. It computes the weighted average over all MSD curves from the particle trajectories. Weights are taken to be the number of averaged delays in individual curves, which favors short delays. The weighted means was calculated as an approximation, using the next formula: weighted std/$(N_{freedom})^{1/2}$. Trajectories were analyzed with MATLAB, using a package specifically developed for MSD analysis, publicly available: http://www.mathworks.com/matlabcentral/fileexchange/40692-mean-square-displacement-analysis-of-particles-trajectories

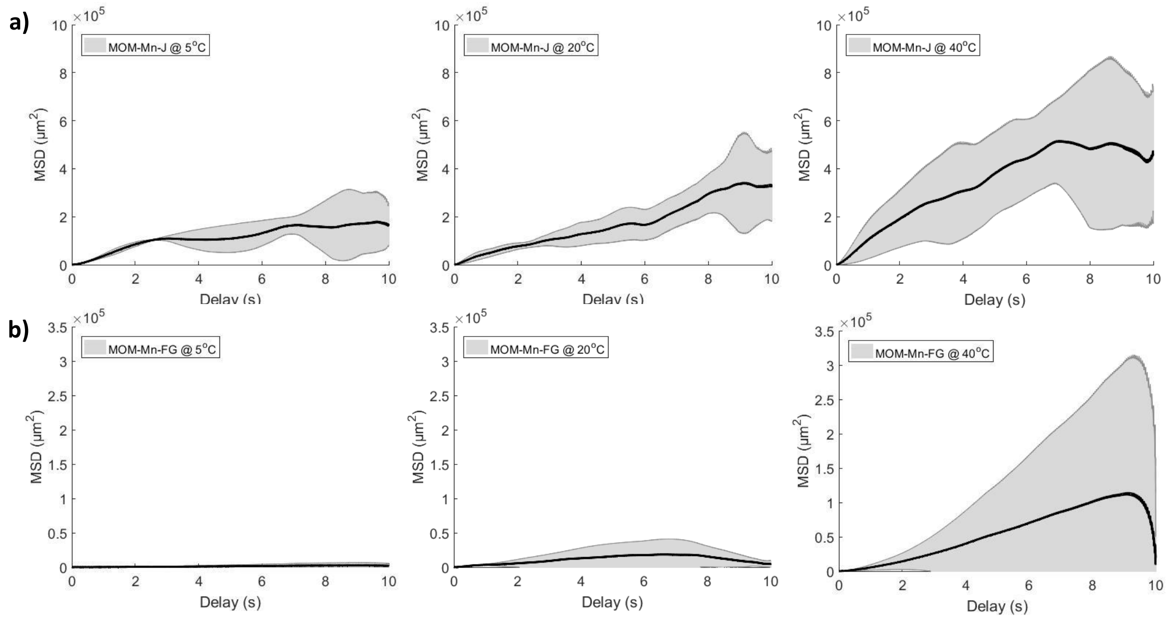

Figure S18. MSD curve for Janus (MOM-Mn-J, a) and fully grafted (MOM-Mn-FG, b) micromotors (Number of particles=4), at 5, 20 and 40 °C. The grayed area represents the weighted standard deviation over all MSD curves.

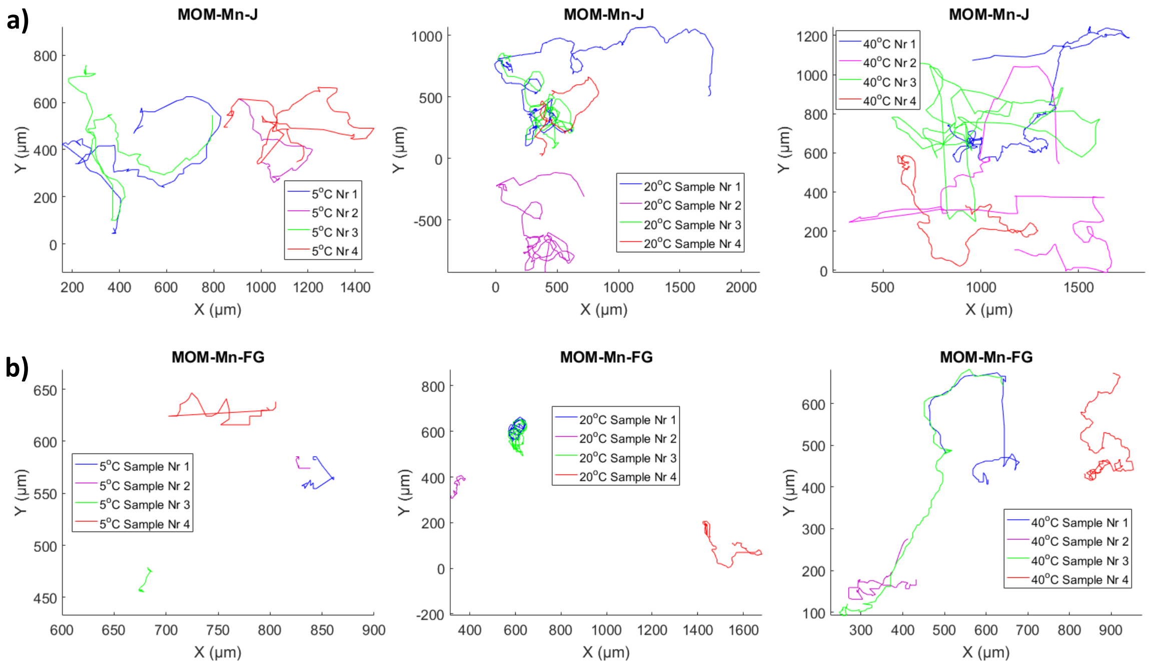

Figure S19. Tracking paths for Janus (MOM-Mn-J, a) and fully grafted (MOM-Mn-FG, b) micromotors (Number of particles=4), at 5, 20 and 40 °C.

Supporting Videos

Supporting Video 1 (SV1). MOM-Mn-J presence of hydrogen peroxide (fuel concentration H_2O_2 1 % v/v), in 1 % SDS, at 40 °C

Supporting Video 2 (SV2). MOM-Mn-J in presence of hydrogen peroxide (fuel concentration H_2O_2 1% v/v) in 1 % SDS, at 40 °C and at 5 °C.

Supporting Video 3 (SV3). MOM-Mn-FG in presence of hydrogen peroxide (fuel concentration H_2O_2 1 % v/v) in 1% SDS, at 40 °C and at 5 °C.

Supporting Video 4 (SV4). MOM-Mn (control) in presence of hydrogen peroxide (fuel concentration H_2O_2 1 % v/v), at decreasing temperatures from 40 to 5 °C.

Supporting Video 5 (SV5). MOM-Mn (control) in presence of hydrogen peroxide (fuel concentration H_2O_2 1 % v/v in 1% SDS), at 35 °C.

References

[1] M. Kneidinger, A. Iturmendi, C. Ulbricht, T. Truglas, H. Groiss, I. Teasdale and Y. Salinas, Mesoporous Silica Micromotors with a Reversible Temperature Regulated On–Off Polyphosphazene Switch. *Macromol. Rapid Commun*. **2019**, *40*, 1900328.

[2] K. Parida, D. Rath, Amine functionalized MCM-41: An active and reusable catalyst for Knovenagel condensation reaction, *J. Mol. Cat. A: Chem*., **2009**, *93*, 93-100.

[3] M. Kapoor, Q. Yang, S. Inagaki, Self-Assembly of Biphenylene-Bridged Hybrid Mesoporous Solid with Molecular-Scale Periodicity in the Pore Walls, *J. Am. Chem. Soc*. **2002**, *124*, 15176- 15177.

[4] J. J. Teesdale, A. J. Pistner, G. P. A. Yap, Y.-Z. Ma, D. A. Lutterman, J. Rosenthal, Reduction of CO_2 using a Rhenium Bipyridine Complex Containing Ancillary BODIPY Moieties, *Catal. Today*, **2014**, *225*, 149-157.

[5] V. López, M. R. Villegas, V. Rodríguez, G. Villaverde, D. Lozano, A. Baeza, and M. Vallet-Regí, Janus Mesoporous Silica Nanoparticles for Dual Targeting of Tumor Cells and Mitochondria, *ACS Appl. Mater. Interfaces*, **2017**, *9*, 26697- 26706.

[6] Y. Salinas, et.al, Biocompatible Phenylboronic-Acid-Capped ZnS Nanocrystals Designed as Caps in Mesoporous Silica Hybrid Materials for on-Demand pH-Triggered Release in Cancer Cells, *ACS Appl. Mater. Interfaces*, **2018**, *10*, 34029- 34038.

[7] Nadine Tarantino, Jean-Yves Tinevez, Elizabeth Faris Crowell, Bertrand Boisson, Ricardo Henriques, Musa Mhlanga, Fabrice Agou, Alain Israël, Emmanuel Laplantine, TNF and IL-1 exhibit distinct ubiquitin requirements for inducing NEMO–IKK supramolecular structures, *J Cell Biol*, **2014**, 204, 231–245.
